# Epidémiologie et facteurs de risque des anomalies de fermeture du tube neural: données marocaines

**DOI:** 10.11604/pamj.2015.22.43.5158

**Published:** 2015-09-17

**Authors:** Mohammed Amine Radouani, Naima Chahid, Loubna Benmiloud, Laila Elammari, Khalid Lahlou, Amina Barkat

**Affiliations:** 1 Service de Médecine et Réanimation Néonatales, Centre National de Néonatologie et Nutrition, Hôpital d'Enfants, Centre Hospitalier Ibn Sina, bd Ibn Rochd, Souissi 10100, Rabat, Maroc; 2 Equipe de Recherche en Santé et Nutrition du Couple Mère Enfant, Faculté de Médecine et de Pharmacie de Rabat, Université Mohammed V, Souissi, Maroc; 3 Ministère de la Santé, Direction de la population, Maroc Service de Médecine et Réanimation Néonatales, Centre National de Néonatologie et Nutrition, Hôpital d'Enfants, Centre Hospitalier Ibn Sina, bd Ibn Rochd, Souissi 10100, Rabat, Maroc

**Keywords:** Anomalies de fermeture, tube neural, épidémiologie, facteurs de risque, Closing anomalies, neural tube, epidemiology, risk factors

## Abstract

**Introduction:**

Les anomalies de fermeture du tube neural sont des défauts congénitaux de la formation du système nerveux central. L'incidence varie entre 3 et 40 cas pour 10000 dans le monde. Il existe des facteurs de risque de survenue de cette affection. La prévention reste un élément important dans la prise en charge. L'objectif de ceete étude est d’étudier les paramètres sociodémographiques, maternels, obstétricaux et néonatals des anomalies de fermeture du tube neural et analyser les facteurs de risque responsables dans notre contexte.

**Méthodes:**

Etude prospective cas-témoin sur 4 ans. Ont été recrutés tous les cas portant une malformation du tube neural isolée ou associée à d'autres malformations. Les données maternelles, obstétricales et néonatales ont été enregistrées. L'analyse statistique était réalisée par le biais d'un logiciel de statistiques SPSS version 17.0 pour Windows.

**Résultats:**

Soixante huit cas ont été inclus. Quatre-vingts cinq pour cent des malformations étaient isolées. L'anencéphalie était l'anomalie la plus retrouvée (67%). L’âge maternel moyen était 31,03±7,50 ans. La consanguinité parentale était notée dans 9 cas. Un niveau socio-économique bas et un non suivi des grossesses ont été rapportés dans 29% des cas. L’étude a retrouvé des antécédents de mort-nés et de morts néonatales dans 4% des cas. La consommation de Fenugrec était significativement associée aux malformations du tube neural et a été retrouvée dans 8 cas contre 1 cas dans le groupe sain. La voie haute d'accouchement était utilisée dans 29% des cas. L’âge gestationnel moyen était de 35,55±4,16 semaines d'aménorrhée. Il n'y avait pas de prédominance de sexe. On avait noté une relation significative entre les malformations du tube neural et l'avènement d'une asphyxie périnatale, 15 cas présentaient un apgar à 0 à la première minute et 12 cas un apgar inférieur à 7 à la cinquième minute.

**Conclusion:**

Le bas niveau socio-économique, le non suivi des grossesses et la consommation maternelle de fenugrec en période gestationnelle étaient des facteurs prédictifs de développement d'anomalies du tube neural dans notre contexte.

## Introduction

Les anomalies de fermeture du tube neural AFTN sont des embryopathies apparaissant pendant les 3^e^ et 4^e^ semaines de la vie intra-utérine et caractérisées par des défauts congénitaux de la formation du système nerveux central. Elles représentent 5% des malformations congénitales. L′origine de ces malformations est multifactorielle, avec interaction des facteurs environnementaux et génétiques. Ces malformations sont responsables le plus souvent de séquelles psychomotrices et sphinctériennes importantes nécessitant une prise en charge multidisciplinaire. La prévention par le conseil génétique, la prise d'acide folique en période péri-conceptionnelle et la précision du diagnostic anténatal ont fait baisser la prévalence des défects du tube neural dans plusieurs pays industrialisés [1]. L'objectif de notre travail est de déterminer la prévalence des AFTN dans notre maternité, d'en déterminer les facteurs sociodémographiques, maternels et obstétricaux associés et d'identifier les facteurs de risque impliqués dans la survenue des AFTN dans notre contexte.

## Méthodes

Nous avons conduit, pendant quatre ans (Janvier 2008 à Décembre 2011) une étude prospective cas-témoin au niveau du centre de référence en néonatologie et nutrition de l'hôpital d'enfants de Rabat en étroite collaboration avec la maternité Souissi de Rabat.

La Maternité Souissi est une maternité de type III, c'est une maternité de référence qui reçoit de toutes les régions du royaume et dont le nombre annuel des accouchements s’évalue à une moyenne de 13000. De ce fait les cas analysés donnent une idée relativement générale sur la pathologie dans le pays.

Nous avons retenu comme cas toute naissance vivante ou mort-né présentant une anomalie de fermeture du tube neurale (AFTN) isolée ou associée à une autre anomalie congénitale. Ont été recrutés comme cas témoins, toutes les naissances survenant le même jour et dans l′heure qui suit la naissance des nouveau-nés avec AFTN. Tous les cas ont été rapportés au nombre de naissances vivantes enregistrées pendant cette période.

Des informations générales sur le nombre des cas malformés chaque année ont été enregistrées ainsi que des données comprenant les antécédents maternels, le niveau socio-économique, la présence d'une consanguinité, le déroulement de la grossesse et de l'accouchement ainsi que les données néonatales.

### Définitions des termes

Dysraphisme spinal ou spina bifida est un ensemble de malformations complexes caractérisées par l'absence de l'arc postérieur sur une ou plusieurs vertèbres. Elles intéressent à un degré divers les enveloppes méningées, la moelle épinière et les racines.

Spina bifida occulta représente la forme mineure et la plus répandue de la malformation. Il n′existe pas d′ouverture ni de déformation cutanée dans la région lombo-sacrée (la peau en regard des anomalies peut rester normale ou porter une zone très poilue, un nævus ou un hémangiome, voire une petite dépression du derme). Encéphalocèle: se définit par une hernie du tissu cérébral et/ou des méninges à travers une déhiscence de la boite crânienne. Spina bifida à moelle ouverte: C′est le cas où, durant la morphogenèse spinale la gouttière neurale ne se développe pas, laissant ainsi un arc neural totalement ouvert. Anencéphalie: défaut de fermeture du tube neural qui se caractérise par l′absence totale ou partielle de voûte crânienne et de cuir chevelu, le cerveau étant absent ou réduit à une masse de taille réduite.

Les données sociodémographiques colligées comprenaient le niveau socio-économique de la famille, le niveau d'instruction de la parturiente et l'existence ou non d'une consanguinité parentale. Les données maternelles étudiées étaient l’âge pendant la grossesse, les antécédents d'avortements ou de mort-nés, l'existence ou non d'un suivi de grossesse et la consommation éventuelle de fenugrec durant la grossesse, ou d'autres plantes médicinales. Les données néonatales étaient représentées par l’âge gestationnel, la voie d'accouchement, le sexe, l'apgar et le type d'AFTN pour les cas. Est dite naissance normale: toute naissance indemne de toute AFTN ou autres malformation congénitale cliniquement visible. Est dite primipare toute femme étant à son premier d'accouchement et multipare celle ayant plus de trois accouchements. Nous avons considéré toute famille dont le revenu journalier est inférieur ou égal à 5 dollars par jour, comme ayant un bas niveau socioéconomique.

### Etude statistique

L'analyse statistique était réalisée par le biais d'un logiciel de statistiques SPSS version 17.0 pour Windows. Les variables quantitatives étaient exprimées en moyenne +/- écart type et celles qualitatives en pourcentage. Les données continues étaient présentées en moyenne. Lorsque les données n'obéissaient pas à une distribution normale, les données étaient rapportées en médianes et en interquartile(IQR). Les données discrètes étaient décrites en fréquence et en pourcentage. Des comparaisons entre les données continues ont été effectuées en utilisant le test t de student. La comparaison des distributions a été effectuée à l'aide du test de Chi2 ou du test exact de Fisher, avec comme seuil de significativité une valeur p < 0,05.

Une analyse de régression logistique a été utilisée afin d’étudier la relation entre les facteurs de risque probables et la survenue d'une AFTN. Les paramètres présentant des corrélations significatives étaient testés par une analyse de régression multivariée.

## Résultats

### Prévalence des AFTN

Entre janvier 2008 et décembre 2011, 68 cas des AFTN ont été inclus. La prévalence moyenne parmi les naissances vivantes avait passé de 21,78 en 2008 à 12,1 cas par 10000 naissances vivantes en 2011. Quatre-vingt cinq pour cent des AFTN étaient isolées. Les malformations retrouvées étaient une anencéphalie (45 cas), un spina bifida (22 cas) et un encéphalocéle (1cas). Les malformations associées aux AFTN étaient une hydrocéphalie (4cas), une macrocranie (1cas), une fente labiopalatine avec malformation des membres et cardiopathie (1cas), un pied bot (1cas), un pied bot avec faciès trisomique (1cas), un pied bot avec anomalie des organes génitaux externes (1cas) et une polymalformation (3cas).

### Analyse des facteurs de risque

La comparaison des cas AFTN aux témoins a été réalisée sur la base des facteurs sociodémographiques, maternels, obstétricaux et néonatals. Pour le groupe AFTN+, Vingt-neuf pour cent parmi eux sont issus de grossesses non suivies. L’âge moyen des mères était de 31,03±7,50. Vingt pour cent des familles avaient un bas niveau socio-économique. Vingt pour cents des cas étaient issues d'un mariage consanguin (Tableau 1). Concernant les antécédents obstétricaux des mères, on avait noté un antécédent de mort-nés dans deux cas et de mort néonatale dans un cas. Dans 8 cas, une consommation de Fenugrec a été observée. L'accouchement par voie basse a été noté dans 70% des cas. Le bas niveau socioéconomique était significativement associé aux AFTN (29% vs 14%, p = 0,03). En analyse univariée, le non suivi des grossesses étaient significativement associé à un risque élevé de développer une AFTN (95% vs 29%, p < 0,05) (Tableau 1, Tableau 2).

**Tableau 1:** Données maternelles

	*Groupe I* *N = 68*	*GroupeII* *N = 100*	*P*
Age de la mère	31,03±7,50	28,90±5,85	0,14
Consanguinité parentale	n = 9	n = 10	0,31
Bas niveau socio-économique	n = 20	n = 14	0.03
ATCD mort néonatale	n = 1	n = 2	0.2
ATCD mort infantiles	n = 0	n = 2	0,30
ATCD mort nés	n = 2	n = 1	0.21
Consommation de Fenu grec pendant la grossesse	n = 8	n = 1	<0,05

Groupe I: avec malformation; Groupe II: sans malformations

**Tableau 2:** Facteurs de risque de survenue d'une AFTN: analyse multivariée

Paramètre	*P Value*
Bas niveau socioéconomique	0,03
Surveillance de la grossesse	<0,05
Consommation de Fenugrec pendant la grossesse	<0,001

Nous n'avons pas observé de différence significative concernant la consanguinité et l’âge maternel entre les groupes AFTN+ et AFTN- (p = 0,25). En analyse univariée, la consommation de Fenugrec constituait un facteur de risque de développer une AFTN (11% vs 1%, p < 0,05 (Tableau 1, Tableau 2).

Concernant les caractéristiques néonatales (Tableau 3), le sex-ratio était équilibré dans les deux groupes. L’âge gestationnel moyen du groupe AFTN + était de 35,55±4,16 semaines d'aménorrhée SA contre 38,67±3,70 pour le groupe témoin. Vingt-deux pour cent des malformés avaient un apgar à 0 à une minute et 17% avaient un apgar <7 à cinq minutes. L'analyse statistique a montré que les AFTN étaient statistiquement corrélées à un apgar à 0 à la première minute et à un apgar inférieur à 7 à la 5ème minute (Tableau 2).

**Tableau 3:** Données néonatales

	*Groupe I* *N = 68*	*Groupe II* *N = 100*	*P*
**Age gestationnel (SA)**	35,55±4,16	38,67±3,70	0,11
**Voie basse**	n = 48	n= 85	0,85
**Voie haute**	n= 20	n = 15	0,85
**Sexe féminin**	n = 38	n = 58	0.82
**Sexe masculin**	n = 30	n = 32	0.82
**Apgar 1’ 0/10**	n = 15	n = 1	<0,05
**Apgar 5’ <7/10**	n = 12	n = 1	<0,05
**AFTN isolée**	n = 58		
**AFTN associée**	n= 10		

Groupe I: avec malformations; Groupe II: sans malformations

## Discussion

Soixante huit cas ont été inclus dans notre série. Quatre-vingts cinq pour cent des malformations étaient isolées. Quatorze pour cent des nouveau-nés étaient issus d'un mariage consanguin. Un niveau socio-économique bas et un non suivi des grossesses ont été rapportés dans 29% des cas. Il n'y avait pas de prédominance de sexe. L’âge gestationnel moyen était de 35,55±4,16 semaines d'aménorrhée. La consommation de Fenugrec durant la grossesse était significativement associée aux malformations du tube neural. Il y'avait une relation significative entre les malformations du tube neural et l'avènement d'une asphyxie périnatale.

L'incidence des AFTN dans notre étude est passée à 1,2/ 1000 naissances vivantes en 2011. L'incidence de ce type de malformation en Inde varie de 0,6 à 13/ 1000 naissances, ce taux varie selon les types de populations. Certains groupes, de descendance galloise, irlandaise et sikh entre autres, présentent un risque d′anomalies du tube neural beaucoup plus élevé que la population générale. L'incidence au Royaume uni, au Danemark et à Oman ne dépasse pas les 1,5/1000 naissances. En Colombie, l'incidence des AFTN est de 1/1000. Aux Etats-Unis, la fréquence est de 1/2000 en moyenne, celle des spina bifida y décroît d′est en ouest, avec un taux minimal de 0,1/1000 au nord de la côte Pacifique [2, 3].

Le taux de récurrence chez les femmes qui ont déjà donné naissance à un enfant atteint s′élève à 2% (20 cas pour 1 000 naissances vivantes), mais dans les populations très vulnérables, le risque de récurrence peut atteindre les 4 à 5% (40 à 50 cas pour 1 000 naissances vivantes) vivantes. Dans notre étude, on n'avait pas noté de récurrence des AFTN chez les mères, Jayesh a rapporté en 2003 en Inde un cas de myélomeningocèle récurrent chez 4 enfants de la même fratrie issus d'un mariage consanguin [3].

Les AFTN sont plus fréquentes chez les f'tus de sexe féminin, ce déséquilibre est plus marqué pour les anencéphalies. Dans notre série, la répartition selon le sexe était équilibrée. La fréquence est plus élevée en cas de grossesse gémellaire selon une cohorte française, mais le plus souvent un seul jumeau est atteint [4]. Le bas niveau socioéconomique est l'un des facteurs de risque les plus fréquemment associés aux AFTN. Notre série a confirmé cette donnée sociodémographique, ceci pourrait être attribué au rôle des carences nutritionnelles maternelles en particulier en acide folique [4, 5].

Le facteur saisonnier a été évoqué dans certaines séries, il existerait même un pic de fréquence en Grande-Bretagne et en Jordanie chez les enfants conçus au printemps [6].

L′anencéphalie était l'AFTN la plus courante dans notre étude, une étude réalisée par Dhapate et al. a montré que l'hydrocéphalie avec spina bifida était la AFTN la plus fréquente. L'hydrocéphalie et l′anencéphalie étaient presque égales dans une étude rapportée par Balakumar [7, 8].

La pathogenèse des AFTN reste encore obscure et controversée. Divers facteurs sont considérés comme facteurs étiologiques, tels que l'hyperthermie, l′acide valproïque, le fenugrec l'hypervitaminose A, la carence en acide folique et en vitamine B12 pendant la période périconceptionnelle et les facteurs génétiques [9, 10]. En 2008, une étude marocaine avait rapporté que la consommation de fenugrec par les femmes enceintes était associée de façon significative à l'avènement des malformations du tube neural chez le nouveau-né [11].

Notre étude confirme, sur ce plan, les données de la littérature concernant la corrélation entre la consommation de fenugrec par les parturientes, la non surveillance des grossesses et le développement d'AFTN.

En Irlande, selon une étude de Mc DONNELL RJ et coll. la prévalence AFTN est passée de 46.9/10.000 naissances à 11.6/10.000 entre 1980 et 1994. Ces auteurs attribuent ces résultats à la prise d'acide folique en période périconceptionnelle [4]. Molloy et coll. rapportent qu'il y a une corrélation entre le taux de folate dans le globule rouge en période périconceptionnelle et les défects du tube neural. Ce taux de folate a un déterminisme génétique. Il s'agit selon ces auteurs du gène C677T. Dans la forme homozygote, le taux de folate érythrocytaire est de 20% inférieure à la normale expliquant ainsi que la prévalence du génotype TT est élevée de façon significative chez les enfants porteurs d'AFTN et chez leurs parents. Dans d'autres études, d'autres gênes seuls ou en interaction sont impliqués dans la survenue des AFTN (PDGFRA, CBS,MS, MTHFR) [12].

En présence d′anomalies du tube neural, le dépistage prénatal au moyen d′un dosage des alphafoetoprotéines sériques maternelles combiné à une échographie permet de détecter entre 85 et 90% des grossesses touchées s′il est effectué entre la quinzième et la vingtième semaine de grossesse ce qui n’était pas le cas chez 70% de nos parturientes [13].

The American Academy of Pediatrics reconnue par l'US Public Health Service (USPHS) recommande la prise de 400 microgrammes d'acide folique par jour en période péri-conceptionnelle. Cette mesure permet de baisser la prévalence des malformations du tube neural de plus de 50%. Pour les mères ayant des antécédents de spina bifida, il est recommandé la prise de 4000UI d'acide folique par jour pendant 3 mois [3, 14].

Dans cette étude 30% des mères ont un niveau socio-économique faible et ne sont pas instruites. Ceci augure les difficultés de communication pour une stratégie préventive. Dans notre contexte donc, la baisse de l'incidence entre 2008 et 2011, pourrait être expliquée par la mise en place en 2008 du programme national de fortification de la farine en acide folique. Néanmoins, cette prévention devrait être renforcée par la prise d'acide folique en période péri-conceptionnelle chez les cas à haut risque et par l’éviction de consommation de Fenugrec notamment durant le premier trimestre grossesse, ainsi que par une surveillance rigoureuse des grossesses notamment à haut risque.

La mortalité varie entre 20 et 36%. Les anomalies du tube neural sont associées à une morbidité considérable. L′anencéphalie n'est pas compatible avec la vie. Les nouveau-nés atteints de spina bifida ont un pronostic différent, si la lésion en regard de la moelle épinière n′est pas fermée à temps, l’évolution est rapide vers le décès en quelques jours, s'il ya une possibilité de fermeture et si l′hydrocéphalie associée est traitée avec un dispositif de dérivation, le pronostic est favorable. Le pronostic à long terme dépend du degré de l′atteinte neurologique, intellectuelle et de la gestion des complications liées à l'hydrocéphalie [4, 5, 7].

## Conclusion

Les malformations du tube neural restent des anomalies fréquentes dans notre contexte. Elles sont responsables d'une mortalité et d'une morbidité importantes. La stratégie de prévention des malformations du tube neural devrait être renforcée en insistant sur le suivi des grossesses, l’éviction de consommation de Fenugrec en période gestationnelle et la supplémentation en acide folique des grossesses à haut risque de récurrence des anomalies de fermeture du tube neural.
